# The inter-organelle cross-talk finely orchestrated in the amyloidogenic processing of amyloid precursor protein in dendritic arborization neurons of *Drosophila*

**DOI:** 10.7150/thno.104345

**Published:** 2025-02-10

**Authors:** Guo Cheng, Jin Chang, Shanshan Ke, Zimin Dai, Deyong Gong, Hui Gong, Wei Zhou

**Affiliations:** 1 Britton Chance Center for Biomedical Photonics, Wuhan National Laboratory for Optoelectronics, MoE Key Laboratory for Biomedical Photonics, Huazhong University of Science and Technology, Wuhan 430074, China.; 2 HUST-Suzhou Institute for Brainsmatics, JITRI, Suzhou 215123, China.

**Keywords:** inter-organelle communication, organelle contact sites, complementary modulation, amyloidogenic processing of APP, Alzheimer's disease, dendritic arborization neurons, *Drosophila*

## Abstract

**Background:** Organelles in neuronal dendrites facilitate local metabolic processes and energy supply, crucial for dendrite development and neurodegenerative diseases. The distinct functions of dendritic organelles have been well studied, however, their crosstalk under physiological and pathological contexts remains elusive. We aimed to establish an* in vivo* model system of contacts between multi-organelles for investigating the modulation of inter-organelle crosstalk in Alzheimer's disease (AD).

**Methods:** A dendrite model of organelle contacts was developed in *Drosophila* neurons using a set of proximity-driven probes and four-color Airyscan super-resolution imaging. The systematic modulations among multiple contact sites (CSs) between organelles were examined by manipulating CS tethers and vesicular transporters. Finally, perturbations of these CSs and the dendrite structure in the amyloidogenic processing of amyloid precursor protein (APP) were evaluated by introducing three stages of the processing in this model system.

**Results:** A dynamic network, interconnected via CSs and organized with multi-organelle contacts, was presented among Golgi outposts, the endoplasmic reticulum, lysosomes, and mitochondria (GELM). The CS modulations were found to encompass both their density and motility. Notably, multi-CSs participated in complementary modulations spanning across different cellular pathways. Furthermore, the CS network was revealed to be progressively disturbed in APP amyloidogenic processing, with upregulations in density and motility extending from single- to multi-CSs. These CS perturbations, along with defects in dendrite structural plasticity, could be partially rescued by knocking down Miro.

**Conclusion:** The elucidation of CS modulation modes in the GELM network model reveals a cascaded dysregulation of organelle crosstalk during APP amyloidogenic processing. It expands the mechanisms of inter-organelle communication and provides novel insights into neurodegeneration in AD pathology.

## Introduction

A whole suite of organelles is present in dendrites 1. They possess distinct dynamic and structural characteristics, support local processes of material and energy metabolism, and contribute to dendrite development and plasticity 2. For instance, Golgi outposts (GOs), which differ from stationary, stacked somal Golgi, shuttle in dendrites for local secretion 3-5; and dendritic mitochondria (Mito), which have a more elongated shape and are less dynamic than axonal ones, supply energy for local translation during plasticity 6, 7. The abnormal morphology and dysfunction of these organelles, such as the disruption of compartment organization and transport of GOs 5 and changes in mitochondrial dynamics and morphology 8, 9, are closely related to neurodegenerative diseases. Each of these organelles has distinct functions, and they are not completely isolated in structure and function. Physical connections of these organelles have been observed, such as the endoplasmic reticulum (ER)-Mito, ER-endosome, and lysosome (Lyso)-Mito contacts 10, 11, but the coordination of these contacts remains unclear in dendrites.

Contact sites (CSs) provide a novel pathway for inter-organelle communication, forming when organelles are in close proximity 12. These CSs facilitate the exchange of metabolites between organelles, and play roles in multiple cellular functions, collectively maintaining cellular homeostasis 13-16. In neurons, recent studies show that the CSs in the soma and axon regulate compartmental lipid metabolism, Ca^2+^ transfer, and organelle translocation, contributing to synaptic plasticity and neurite outgrowth 17-19. The dysregulation of CSs with diverse modes has been found in neurodegenerative diseases. For example, the ER-Mito CSs can be down-regulated by mutations in LRRK2 or α-synuclein 20, 21, while up-regulated by the knockout of PINK1 or parkin in Parkinson's disease (PD) 22-24; upregulation of these CSs is also detected in Alzheimer's disease (AD) induced by the products of APP sequential cleavage in amyloidogenic processing, including its C-terminal fragments (APP-CTFs) generated by β-secretase 25 and β-amyloid peptide (Aβ) produced by further γ-secretase cleavage 26, 27. This suggests that understanding of the modulation mode of inter-organelle contacts can help assess their contribution to diseases.

Diverse modulations of CSs, reported from single and pairs of organelle-related CSs, suggest that there may be interconnectivity among different inter-organelle contacts. The uniform modulation of multiple CSs can be mediated by individual CS tethers 28. For instance, multiple CSs on ER can be regulated by the ER protein VAP, which tethers organelles, such as Mito, endosomes, and the Golgi, with the ER 29. Meanwhile, different CSs coordinate in a compensation mode to adapt to the physiological conditions. For example, the depletion of NPC1 downregulates contacts between ER and Lyso but upregulates contacts between Lyso and Mito 30. Currently, the organelle connection network is raised by observations of the contacts in complexes of multiple organelles 10, 31, 32. Moreover, using FIB-SEM imaging and multi-omics analysis, Lee et al. found that dysfunction of any one of the ER, Golgi apparatus, and peroxisomes can trigger abnormalities of multiple organelles in their biogenesis and interactions, leading to global shifts in cellular lipid and protein homeostasis 33. These findings suggest that organelles are in a communicome with high connectivity and interdependence 34, and therefore, the modulations and functions of their communication should be thought at the network level.

Neuronal dendrites, with their branching properties 35, provide a unique model for studying inter-organelle communication at CSs. Compared to the crowded of organelles in the cell body, organelles distributed along the dendrite axis exhibit spatial separation, which enables the accurate observation of organelle contacts in two-dimensional space 11. Moreover, in contrast to the imaging challenges of cells in opaque biological tissues 36, 37, the dendritic arborization (da) neurons in *Drosophila* larvae can be imaged *in vivo* at high resolution with the confocal microscopes due to their location under the transparent epidermis. They provide an opportunity to investigate the modulation and functions of inter-organelle contacts in dendrites *in vivo*. Importantly, da neurons are a classical model for studying dendrite development within the complete neuronal circuitry under physiological and pathological conditions, by combining genetic labeling with *in vivo* imaging 38, 39.

In this study, we present a contact network among the GOs, ER, Lyso and Mito in dendrites of *Drosophila* class Ⅲ da (C3da) neurons, and investigate the modulation modes of their CSs under physiological and pathological conditions. Four types of dynamic CSs between the four organelles were characterized by constructing the proximity-driven probes. Meanwhile, their spatiotemporal organization in dendrites was demonstrated by four-color *in vivo* imaging. Furthermore, the diverse modulation modes of the CS network were elucidated by manipulating the CS tethers or vesicular transporters. Then, the modulation of the CS network and dendrite structural plasticity at distinct stages of amyloidogenic processing were studied to elucidate the pathogenesis of AD.

## Results

### Distinct CSs between organelles organized in dendrites

**Figure 1:**
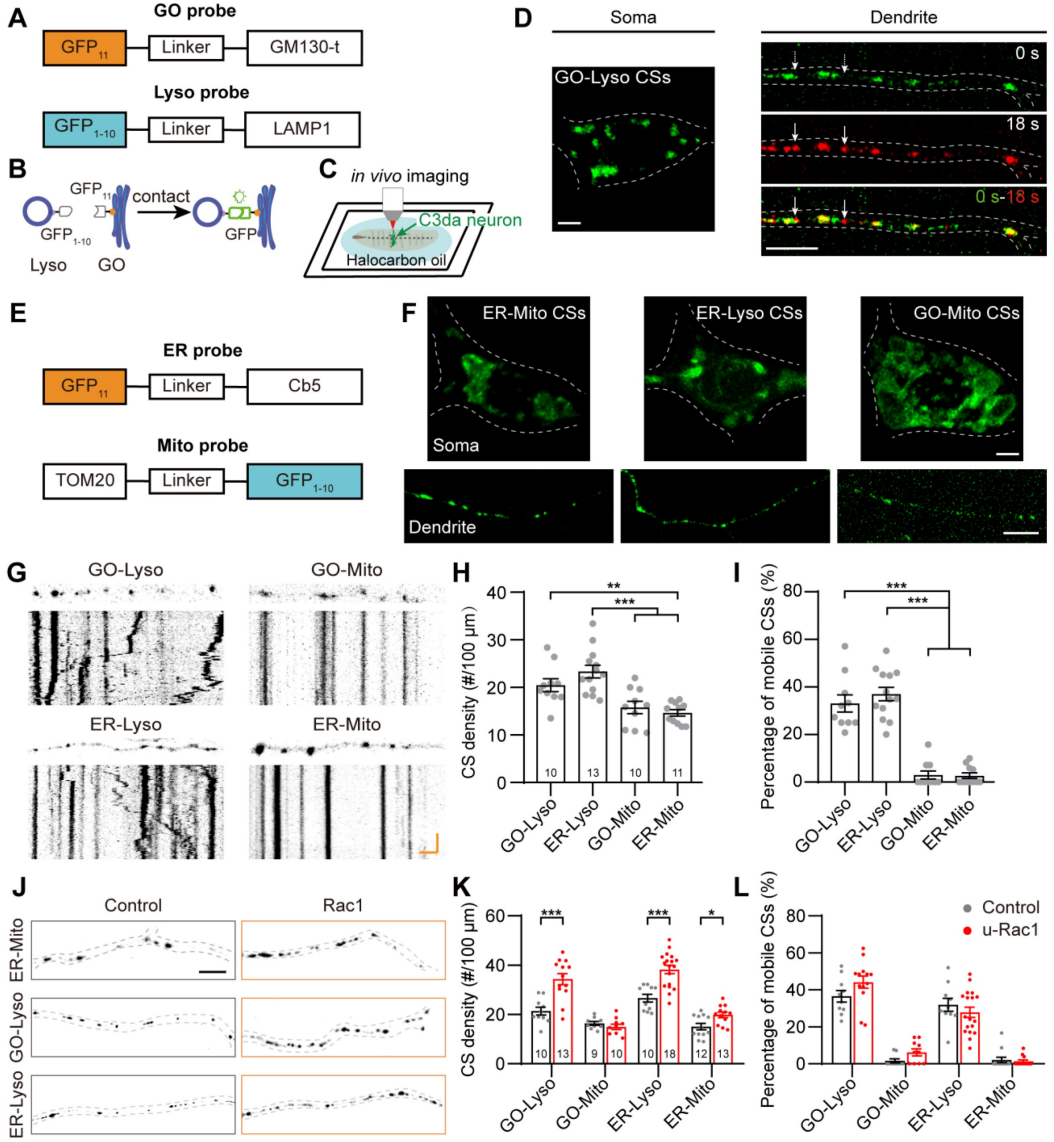
** Four types of CSs in dendrites detected with split-GFP probes. (A, B)** Diagrams of the design of split-GFP probes (A) for labeling GO-Lyso CSs (B). **(C)** Setup for detecting CSs in C3da neurons *in vivo*. **(D)** Representative images showing that GFP reconstituted in C3da neurons and presented as mobilizable puncta in dendrites, when expressing GFP11 and GFP1-10 at GO and lysosomal membranes, respectively. The dynamic puncta in dendrites were shown by merging images from two time points: the initial time point (green) and 18 s later (red). The white arrows indicate the motile puncta. **(E)** Schematic diagram of split-GFP probes for labelling ER with GFP11 and Mito with GFP1-10. **(F)** Confocal images showing the CSs of ER-Mito, ER-Lyso and GO-Mito labelled by reconstituted GFP in soma and dendrites of C3da neurons. **(G)** Representative images of the four types of CSs in straightened dendrites (upper in each type) and the corresponding kymographs (bottom in each type) obtained from time-lapse imaging. **(H, I)** Quantification of the density (H) and motility (I) of the four types of CSs. **(J)** Representative confocal images showing the increased ER-Mito, GO-Lyso and ER-Lyso CSs by the ectopic expression of Rac1. **(K, L)** Quantitative analysis of the effects on the density (K) and motility (L) of the four types of CSs by Rac1. The numbers in the bar diagrams represent the sample sizes of each experimental group from four to six *Drosophila* larvae. For all quantifications, data are the means ± SEM. One-way ANOVA multiple comparisons test with Tukey correction in (H) and (I), and unpaired two-sided Student's t-test in (K) and (L). *p < 0.05, **p < 0.01, ***p < 0.001. Scale bars: 2 μm in soma and 10 μm in dendrites in (D) (F) and (J), kymograph horizontal bar: 2 μm, vertical bar: 1 min in (G).

To visualize the CSs between organelles in dendrites, the split-GFP based proximity-driven probes (split-GFP probes) were chosen because they can detect the distance between organelles within 30 nm in living cells 40-42. We first constructed probes targeted to GOs and Lyso, in which GFP_11_ and GFP_1-10_ were respectively tagged to the cytoplasmic sides of the organelle membranes (Figure 1A). The complete GFP protein would be reconstituted when GOs and Lyso were in close proximity (Figure 1B). CSs labeled by reconstituted GFP signals were observed as movable puncta in dendrites when co-expressing the complementary split-GFP probes in C3da neurons (Figure 1C-D). Further, these CSs were confirmed by assessing the proximity detection, positioning, and fluorescence leaking of the probes. To confirm the sensitivity of the probes to proximity, another type of proximity-driven probes based on fluorescence resonance energy transfer (FRET) were constructed, replacing the two GFP fragments with donor (EGFP) and acceptor (mCherry) fluorophores (Figure S1A-B). The FRET signals were detected at the sites of colocalization of the GO- and Lyso-targeted FRET probes (Figure S1C-D). Then, the positional accuracy of GFP reconstitution was examined by co-expressing the split-GFP probes with GO and Lyso markers. It was found that both stationary and mobile reconstituted GFP signals were colocalized with the GO and Lyso markers, with an 81.4% reconstitution rate on overlapping organelles (Figure S1E-F). Finally, the leakage of probes was checked by expressing the GFP_11_ or GFP_1-10_ probe alone, and by co-expressing GFP_11_-GO with translocated GFP_1-10_ (located in the lumen instead of the outside of the membrane). GFP fluorescence was undetectable under above three conditions (Figure S1G-H), indicating that the probes worked through reconstitution on the cytoplasmic side of adjacent organelle membranes. Together, these results validate that the split-GFP strategy can be used to detect CSs in dendrites* in vivo*.

To further investigate the diversity of CSs in dendrites, we constructed probes targeted to ER and Mito (GFP_11_-Cb5 and GFP_1-10_-TOM20, respectively) (Figure 1E). The ER-Mito, ER-Lyso and GO-Mito CSs were detected by co-expressing the complementary probes (Figure 1F). Then, the distribution and dynamic characteristics of these four types of CSs were evaluated. They all appeared as puncta in dendrites, differed from that in the soma: GO-Lyso, ER-Lyso and ER-Mito CSs were shown as large puncta, while the GO-Mito CSs had ring-like shapes (Figure 1F). The density of these CSs showed different in dendrites: the two CSs related to Lyso (GO-Lyso and ER-Lyso) had higher densities than those related to Mito (GO-Mito and ER-Mito). Their dynamic characteristics were examined by motility. It was found that more than 30% of the GO-Lyso and ER-Lyso CSs were mobile, whereas GO-Mito and ER-Mito ones rarely moved (Figure 1G-I). Besides, the organizational pattern of the CS was stable by assessing their density and dynamics from the second to third instar larvae (Figure S2).

Previous studies have shown the roles of the cytoskeleton in organizing organelles and their contacts 31, 43. We examined whether these CSs labeled with split-GFP probes could be regulated by the ectopic expression of Rac1, which is a regulator of actin cytoskeleton 44. As previous reports 45, 46, the regulation of actin by Rac1 manipulated structural plasticity of dendritic spiked protrusions in da neuron (called “dendritic spikes”) 47 (Figure S3). Then, the density and motility of the four typed of CSs were evaluated. The results indicated a significant increase in the density of three types of CSs except for the GO-Mito. Specifically, the density of GO-Lyso, ER-Lyso and ER-Mito increased from 21.40 to 34.39 sites/100 μm, 26.63 to 38.27 sites/100 μm, and 15.13 to 19.29 sites/100 μm, respectively (Figure 1J-K). And there was no significant difference in their motility (Figure 1L). So, the multi-CSs labeled with split-GFP could be modulated in density by the ectopic expression of Rac1, suggesting the reorganization of CSs in dendrites under the manipulation of the cytoskeleton.

In summary, we establish an imaging model of contacts between multi-organelles using the organelle-targeted split-GFP probes, which characterize the distribution and dynamics of multiple types of CSs between GOs, ER, Lyso and Mito in dendrites.

### A GO-ER-Lyso-Mito network (GELM) predominated by multi-organelle contacts

**Figure 2:**
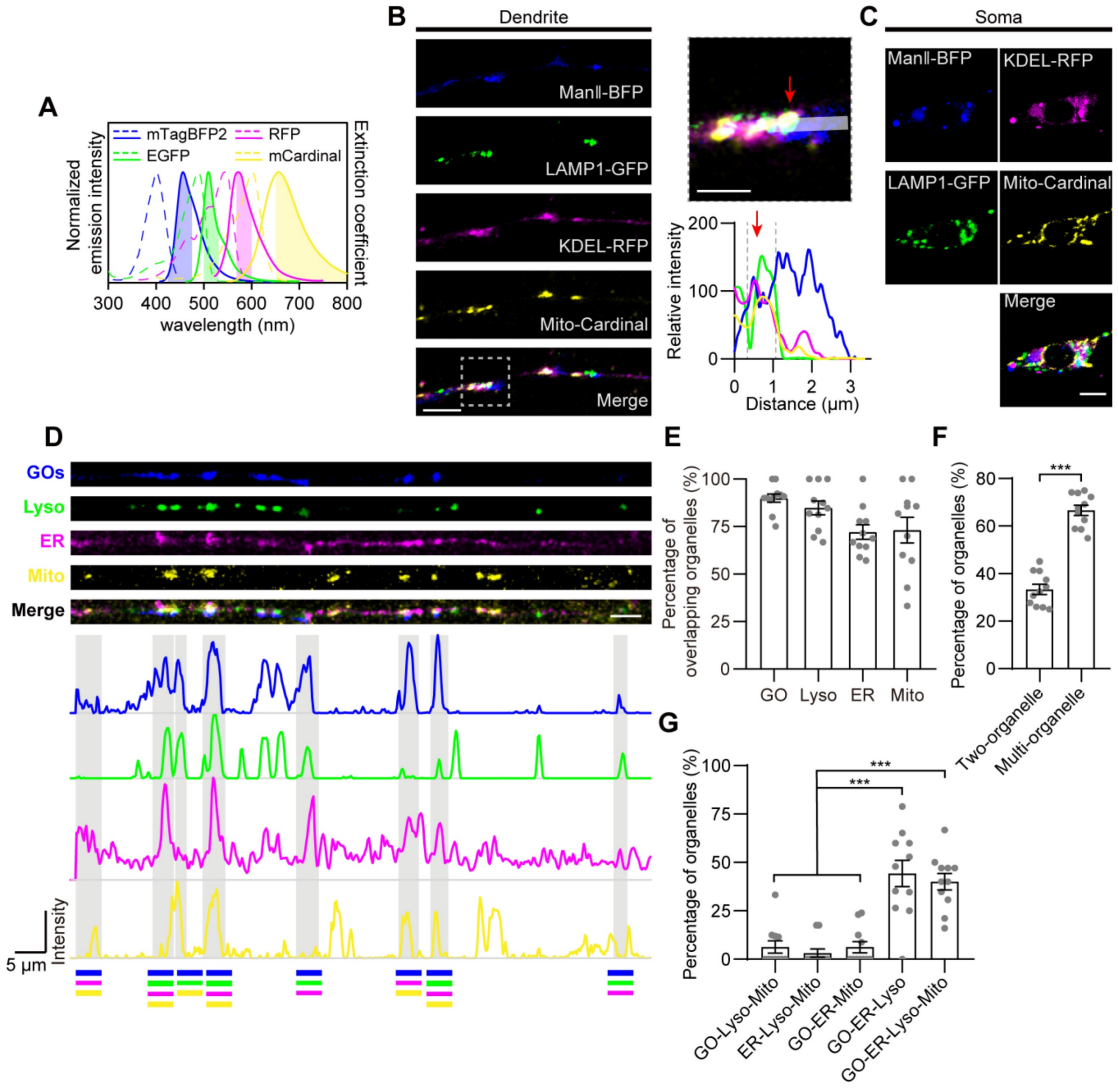
** The spatial organization of GOs, ER, Lyso and Mito in dendrites. (A)** Normalized excitation and emission spectra of the fluorophores used in this experiment: mTagBFP2, EGFP, RFP, and mCardinal. The shaded regions represent the wavelength ranges of detection. **(B, C)** Acquired Airyscan images showing the distribution of GOs (blue), ER (magenta), Lyso (green) and Mito (yellow) in dendrites (B) and the soma (C). Image in (B, right) indicates the magnification of the dotted boxed area in (C, left), and fluorescence intensity profiles were generated along the white line. Red arrows indicate the overlapping site of four organelles. **(D)** Example showing the various spatial overlaps among the four organelles in dendrites. A straightened dendrite with fluorescently labeled organelles (top) and the corresponding fluorescence intensity profiles of the four channels. Overlaps between organelles in distinct complex are shown in gray background. Lines at bottom with different colors represent the organelles in complex: blue for GOs, green for Lyso, magenta for ER, and yellow for Mito. **(E-G)** Quantitative analysis the spatial overlaps among the four organelles. (E) Proportion of each type of organelle overlapping with others. (F) Proportion of organelles in complex of two- and multi-organelles. (G) Proportion of each type of multi-organelle complex. 11 neurons from four *Drosophila* larvae were analyzed. For all quantifications, data are the means ± SEM. Unpaired Student's t-test in (F), and one-way ANOVA multiple comparisons test with Tukey correction in (G). ***p < 0.001. Scale bars: 5 μm in (B, left), (C), and (D), 2 μm in (B, right).

To investigate the spatiotemporal organization of these organelles in dendrites, we performed the multicolor imaging of the four organelles utilizing confocal microscope with Airyscan super resolution module, which can achieve a lateral resolution of 140 nm. Organelles were stably labeled with spectroscopically isolated fluorescent proteins, which are ER (KDEL-RFP), GOs (ManⅡ-mTagBFP2), Lyso (LAMP1-GFP) and Mito (Mito-mCardinal). These four chosen fluorescent proteins, with the distinct emission wavelength spectra, effectively eliminated signal crosstalk and allowed the simultaneous observation of the four organelles in C3da neurons through four-color *in vivo* imaging (Figure 2A, Figure S4). The imaging showed that the four organelles had partial spatial overlaps, which reflected their contacts (Figure 2B-C).

To characterize the contacts among the four types of organelles, we investigated the spatial overlapping at static and dynamic states. The structural images showed that most of the overlap was observed in complexes of three to four organelles, rather than being limited to organelle pairs (Figure 2D). The vast majority of these organelles overlapped with each other, accounting for 89.9% in GOs, 72.1% in ER, 84.8% in Lyso, and 73.1% in Mito, although the four organelles were sparsely distributed in dendrites (Figure 2E). Among them, 33.4% overlapped with only one organelle, while 66.6% were present in multi-organelle complexes, including two main types: GO-ER-Lyso and GO-ER-Lyso-Mito, which accounted for 44.2% and 40.0%, respectively (Figure 2F-G). These results suggest that these four types of organelles prefer to form multi-organelle contacts at spatial positions. Meanwhile, the stability of the organization patterns was assessed by examining the variance in the number of overlaps between organelles over time, considering the dynamic nature of organelles in dendrites (Figure S5A-B). The transient overlap between the four organelles was analyzed through 10-min time-lapse *in vivo* imaging. It showed that the total number of overlaps among them, as well as the overlap number for each organelle pair, remained stable with the average coefficient of variation of 7.9% (Figure S5C), suggesting that their overall organization pattern was steady. These results confirm that the contacts occur frequently and stably, despite the dispersed distribution and dynamic nature of these organelles in dendrites.

Together, the overall organizational connectivity among organelles in dendrites further suggests a GO-ER-Lyso-Mito network (GELM) in dendrites, which possesses a homeostatic spatial-temporal organization pattern with multi-organelle contacts.

### Complementary density modulation between mitochondrial and non-mitochondrial CSs mediated by mitochondrial proteins

**Figure 3:**
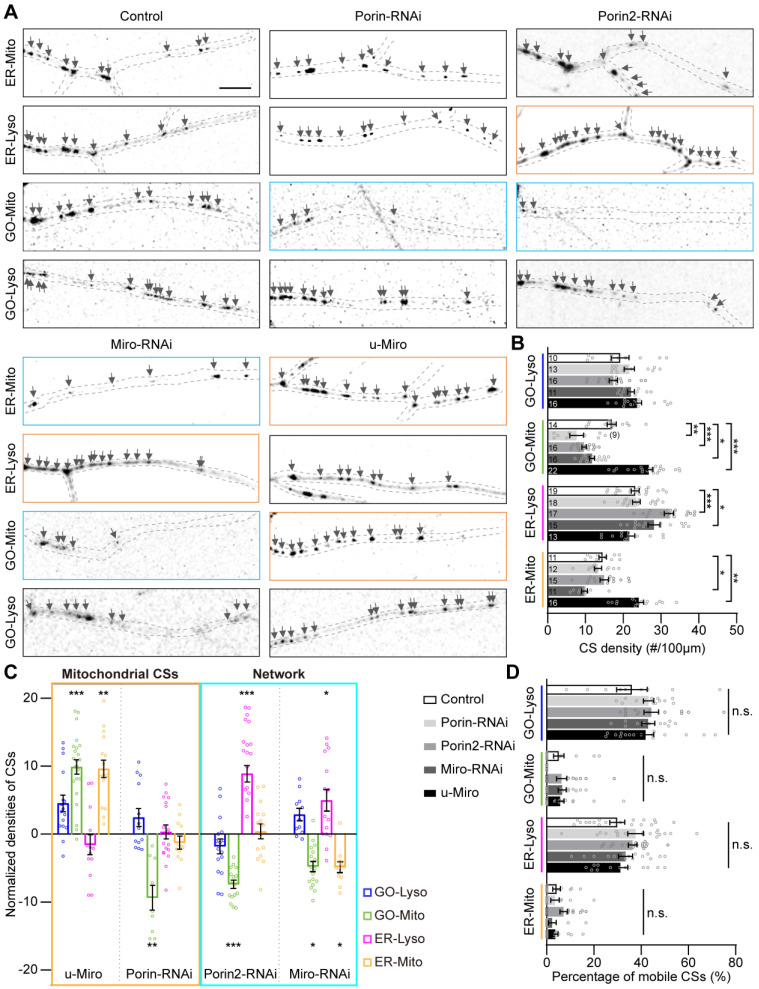
** Modulations of the GELM CSs by mitochondrial CS tethers. (A)** Confocal images of the ER-Mito, ER-Lyso, GO-Mito and GO-Lyso CSs in wild-type neurons and neurons with the manipulation of putative CS tethers. CSs were labelled by split-GFP probes. Arrows indicate the CSs. Dendrites in cyan boxes show decreases in density of CSs and those in orange boxes show the increases. **(B)** Quantitative analysis of the densities of four types of CSs in dendrites with the manipulation of CS tethers. **(C)** Normalized densities of CSs in (B). The CS modulations in Mito-specific and network modes are represented with orange and cyan boxes, respectively. **(D)** Quantitative analysis of the CS motilities with the manipulation of CS tethers. The numbers in the bar diagram represent the sample sizes of each experimental group from four to seven *Drosophila* larvae. For all quantifications, data are the means ± SEM. One-way ANOVA multiple comparisons test with Dunnett correction in (B), (C) and (D). n. s., not significant, *p < 0.05, **p < 0.01, ***p < 0.001. Scale bar: 10 μm.

To further investigate the coordination among the multi-CSs in the GELM network, we examined the modulations of mitochondrial CS tethers on the density of multi-CSs. Given the diversity and versatility of tethers in individual CSs, three mitochondrial proteins (Miro, Porin, and Porin2) were manipulated, which are known as CS tethers in ER-Mito CSs 48, 49. We first evaluated their effects on the two mitochondrial CSs. Consistent with the regulation of CS tethers to their specific CSs 28, the density of mitochondrial CSs was downregulated when knocking down the above three genes by RNA interference (RNAi) (Figure 3A-B). However, there were distinct effects on the GO-Mito and ER-Mito CSs. The GO-Mito CSs were regulated by knockdowns of three proteins, whereas only ER-Mito CSs was reduced by Miro-RNAi. In addition, densities of the two mitochondrial CSs were increased by the ectopic expression of Miro, further confirming the role of Miro in these CSs (Figure 3A-B). These results validate the diverse functions of these CS tethers in the dendritic CSs in C3da neurons.

Then, to figure out the systematic modulations of these CS tethers on the GELM network, the density of the other two CSs (GO-Lyso and ER-Lyso) were also evaluated. Interestingly, Miro and Porin2 also regulated the non-mitochondrial CSs. Contrary to mitochondrial CSs, there was an increase in the density of ER-Lyso CSs in both Miro-RNAi and Porin2-RNAi (Figure 3A). The density of ER-Lyso CSs were from 23.2 to 28.2 sites/100 μm in Miro-RNAi, and from 23.2 to 32.1 sites/100 μm in Porin2-RNAi (Figure 3B). Such two-way modulation between mitochondrial and non-mitochondrial CSs suggests the complementary feedback effects among the GELM CSs (Figure 3C). In addition, these tethers had no effects on the motility of CSs (Figure 3D), indicating that they regulated CS density, rather than motility.

Together, these results reveal that mitochondrial tethers modulated the density of GELM CSs in distinct modes. Especially, the complementary modulation between the mitochondrial- and non-mitochondrial CSs, implies the intricate interactions among CSs existing in the GELM network.

### Vesicular transporters orchestrate the density and motility of the GELM CSs across cellular pathways

**Figure 4:**
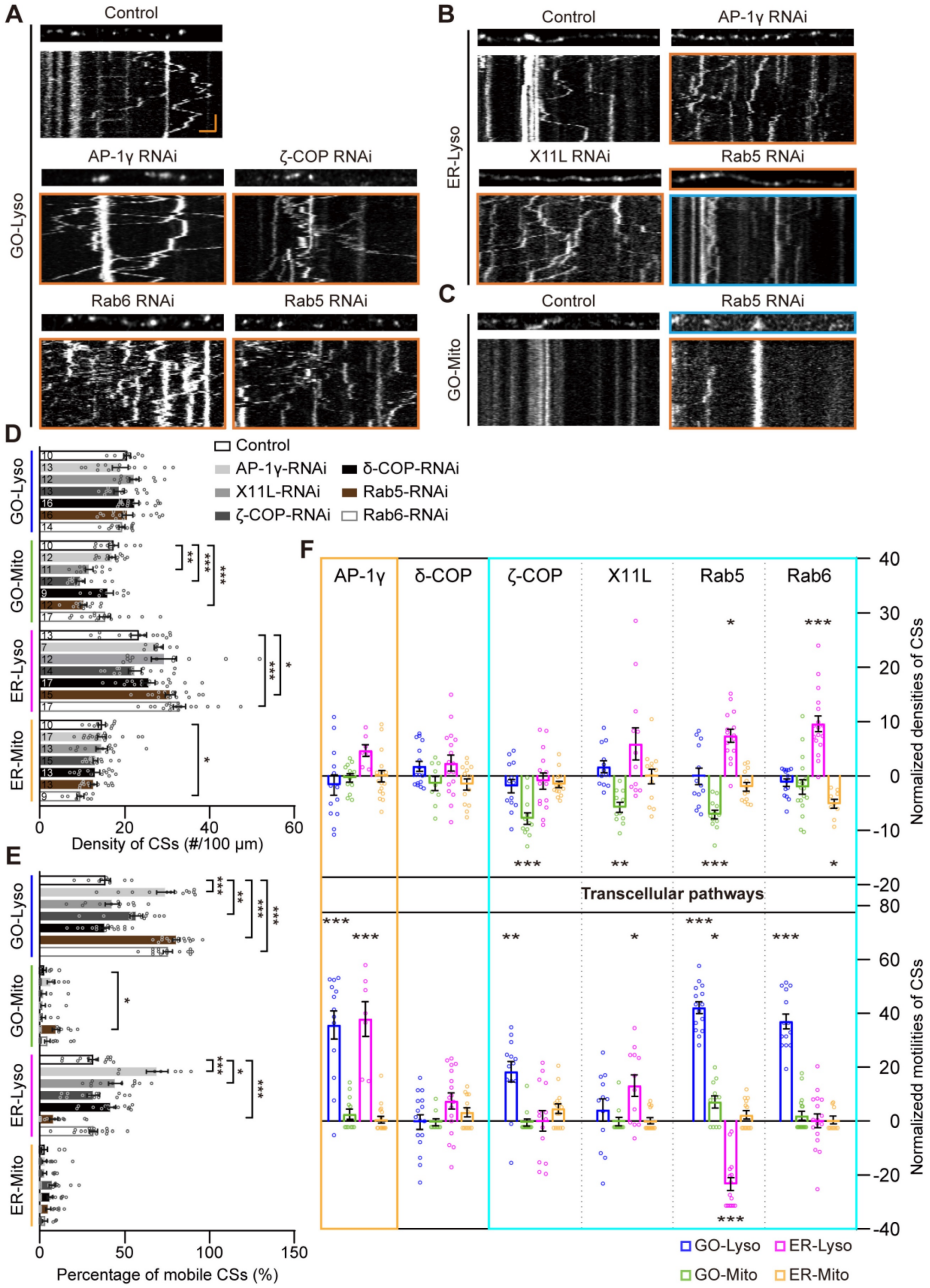
** Modulations of the GELM CSs by the vesicular transporters. (A-C)** Representative confocal images and the corresponding kymographs showing the distribution and movement of GO-Lyso (A), ER-Lyso (B) and GO-Mito (C) CSs in wild-type dendrites and after knockdown of vesicular transporters. CSs were labelled by split-GFP probes. Dendrite in cyan box shows a decrease in CS density and those in orange boxes show the increases. Kymograph in cyan box shows a decrease in CS motility and those in orange boxes show the increases. **(D, E)** Quantitative analysis of the CS densities (D) and motilities (E) in dendrites with knockdown of vesicular transporters. **(F)** Normalized CS densities and motilities in (D) and (E). The modulations of transcellular pathways are represented with cyan box. The numbers in the bar diagram represent the sample sizes of each experimental group from three to six *Drosophila* larvae. For all quantifications, data are the means ± SEM. One-way ANOVA multiple comparisons test with Dunnett correction in (D), (E) and (F). *p < 0.05, **p < 0.01, ***p < 0.001. Horizontal scale bar: 4 μm and vertical bar: 2 min.

Intracellular trafficking is the canonical transport pathway among secretory organelles, including GOs, ER and Lyso, and plays a key role in the regulation of CS-related communications 50, 51. The modulation of the four types of CSs by intracellular trafficking was examined using RNAi knockdown of several typical vesicular transporters, including AP-1γ, δ-COP, ζ-COP, X11L, Rab5 and Rab6 52. It was found that, unlike the density modulation by mitochondrial CS tethers, CS motility could be influenced by these vesicular transporters. Specifically, the motility of GO-Lyso was regulated by AP-1γ, ζ-COP, Rab5 and Rab6; ER-Lyso was mediated by AP-1γ, X11L, and Rab5; and GO-Mito was manipulated by Rab5 (Figure 4 A-C). Moreover, a unique complementary mode between motility and density was found among CSs. For instance, when ζ-COP, X11L, Rab5 or Rab6 was knocked down, a decrease in the density of one type of CS was accompanied by an enhancement in the motility of another type. In particular, Rab5 exhibited two-way modulation in both density and motility: the motility of GO-Lyso and GO-Mito sites increased (from 38.5% to 78.2%, and 2.3% to 9.4%, respectively), but that of ER-Lyso sites decreased (from 31.3% to 8.0%); meanwhile, the density of ER-Lyso sites increased (from 23.2 to 30.6 sites/100 μm) whereas that of GO-Mito sites decreased (from 17.3 to 10.2 sites/100 μm) (Figure 4D-E). These results indicate that the motility of the GELM CSs can be controlled by vesicular transporters, and exhibits a trade-off with CS density.

Intriguingly, it showed that vesicle transporters could regulate Mito-related CSs, but there is no direct link between them. Four of the studied proteins (ζ-COP, X11L, Rab5 and Rab6) modulated the CSs intra-secretory organelles, as well as the CSs between secretory organelles and Mito. Notably, the modulations by X11L, Rab5 and Rab6 implicated CSs on all four types of organelles: GO-Mito and ER-Lyso were affected by X11L-RNAi; GO-Lyso, GO-Mito and ER-Lyso by Rab5-RNAi; and GO-Lyso, ER-Lyso and ER-Mito by Rab6-RNAi (Figure 4F). These results show that modulation of the CSs, mediated by vesicular transporters, can occur across different cellular pathways, further suggesting the network interactions among the GELM CSs.

Thus, vesicular transporters modulate both the motility and density of the GELM CSs, which present a complementary mode across cellular pathways.

### Multi-CSs network progressively implicated in the amyloidogenic processing of APP

**Figure 5:**
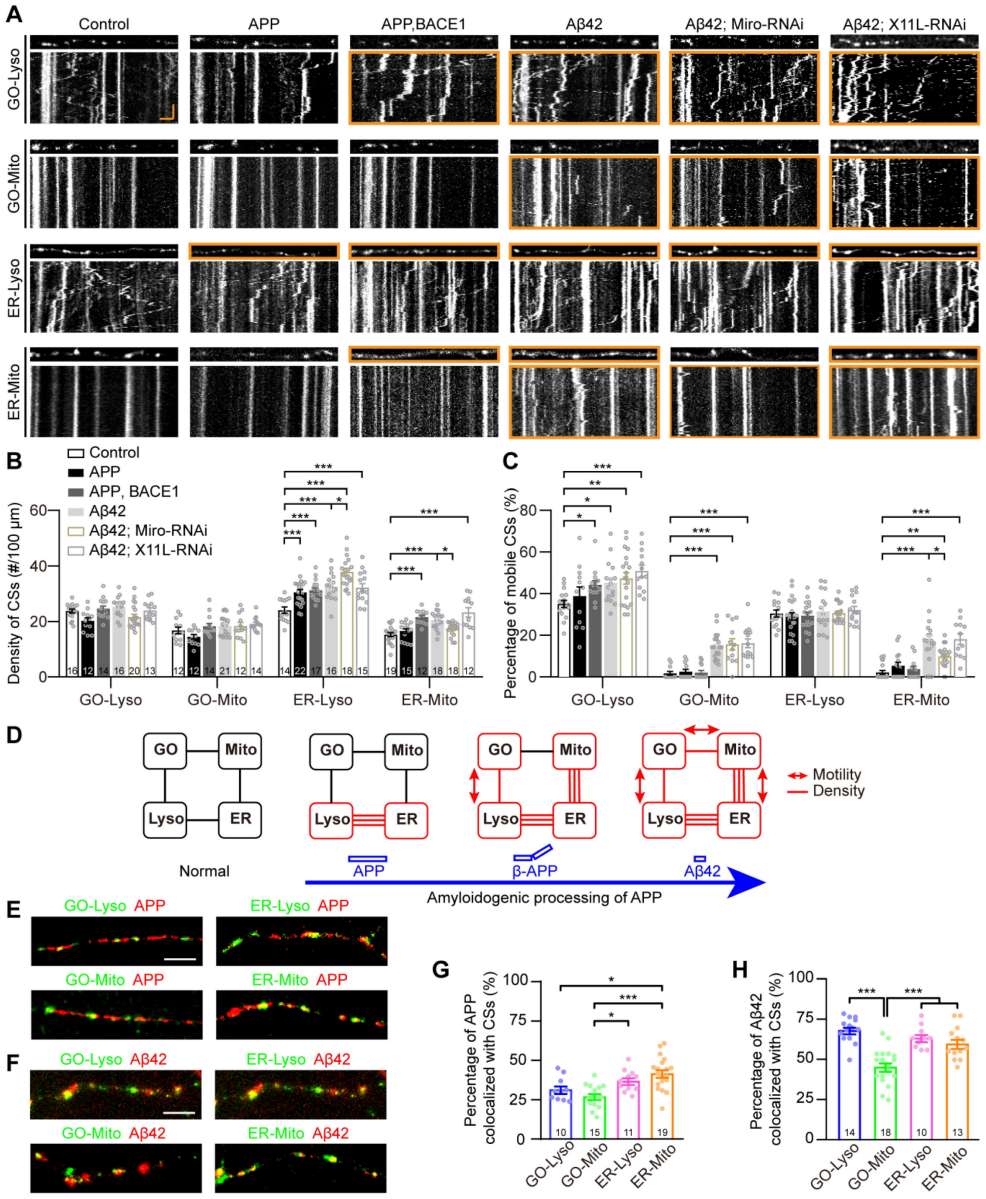
** Progressive disturbances of the GELM CSs in the amyloidogenic processing of APP. (A)** Representative confocal images and the corresponding kymographs showing the distribution and movement of four types of the GELM CSs in the normal, APP, β-APP, and Aβ42 neurons, as well as Aβ42 neurons with the knockdown of Miro, and X11L. Dendrites and kymographs in orange boxes show the increases in CS density and motility, respectively. **(B, C)** Quantitative analysis of effects on CS densities (B) and motilities (C) in the amyloidogenic processing of APP, and the rescue effects of the Miro and X11L knockdown in Aβ42 neurons. **(D)** Diagram showing the modulation mode of the four types of CSs in the amyloidogenic processing of APP. Rectangle boxes indicate organelles, with the red ones indicating organelles on which CSs were altered. Lines between them indicate the CSs between them, with the red lines represent the increase of CS density, and the red lines with bidirectional arrows represent the increase of CS motility. **(E, F)** Representative confocal images showing the colocalization of APP (E, red, mOrange2-APP) and Aβ42 (F, red, Aβ42-TagRFP) with the four types of CSs (green). G, H Quantitative analysis of the colocalization of APP **(G)** and Aβ42 **(H)** with the CSs. The CSs were labelled by split-GFP probes in (A), (E) and (F). The numbers in the bar diagrams represent the sample sizes of each experimental group from four to seven *Drosophila* larvae. For all quantifications, data are the means ± SEM. One-way ANOVA multiple comparisons test with Holm-Sidak correction in (B) and (C), and with Tukey correction in (G) and (H). *p < 0.05, 98 **p < 0.01, ***p < 0.001. Horizontal scale bar: 4 μm and vertical bar: 2 min.

The amyloid plaques, primarily composed of Aβ42, are the pathological hallmark of AD 53. They are generated from the amyloidogenic processing of APP, which has been demonstrated to be associated with the dysregulation of CSs, such as ER-Mito and ER-Lyso 54, 55. In various *Drosophila* AD models, the flies exhibit well-defined neurodegeneration phenotypes, such as synaptic and neuroanatomical defects, reduced locomotion, shorter lifespans, by introducing key amyloidogenic stages in neurons, including the initial full-length human APP695 (APP) 56, the β-cleavage of APP (β-APP, by co-expressing APP695 with β-secretase BACE1) 57, and Aβ42 58, 59. To further investigate whether the GELM network is associated with the organism's pathological status, the four GELM CSs were monitored in neurons undergoing these key amyloidogenic stages. The results showed that upregulation of CSs occurred across all stages, exhibiting a progressive pattern among the four types of CSs and their properties as the amyloidogenic stages progressed from APP to β-APP and subsequently to Aβ42. In detail, there was a significant increase in the density of ER-Lyso in all three amyloidogenic stages: APP, β-APP and Aβ42. As the amyloidogenic stages progresses, the more types of CSs and their properties were changed. For example, the density of ER-Mito and the motility of GO-Lyso also increased significantly in the β-APP stage, while the motility of GO-Mito and ER-Mito also increased in the Aβ42 stage (Figure 5A-C). These results demonstrate the progressive modulation of the GELM CSs in density and motility during the amyloidogenic processing of APP, suggesting a fine and sensitive responsiveness of the dendritic GELM network to the amyloidogenic processing (Figure 5D).

To explore the molecular mechanisms underlying the CS regulation in APP amyloidogenic processing, the rescue phenotypes of the GELM network were also tested with Miro-RNAi, which has been reported to regulate the number of ER-Mito CSs 60. The results showed that Miro-RNAi could partially rescue on the GELM network in Aβ42 neurons by evaluating density and motility of the four CSs. Specifically, Miro-RNAi can completely rescue the density of ER-Mito, and partially rescued the motility of ER-Mito, but could not alter the motility of GO-Lyso and GO-Mito. And more, it accelerated the increase in density of ER-Lyso CSs (Figure 5A-C). These results indicate that Miro-RNAi induces opposing modulation on two types of CSs in Aβ42 neurons, with the phenotype improvement in ER-Mito and worsening in ER-Lyso, suggesting the contributions of the GELM CSs in AD pathologies with diverse modulations. In addition, X11L showed no effect on the CSs in Aβ42 neurons as a control (Fig. 5A-C). Taken together, these results confirm that Miro is involved in CS regulation during APP amyloidogenic processing.

To further confirm the association between AD-related proteins and these CSs, we examined the localization of APP and Aβ42 at the four types of CSs in dendrites. The results showed that both of them could be distributed in the four types of CSs, but the proportion of co-localization was different (Figure 5E-F). The co-localization ratios of APP to GO-Lyso, GO-Mito, ER-Lyso, and ER-Mito, were 31.3%, 26.9%, 36.6%, and 41.3%, respectively. Among them, the proportions of GO-Lyso and GO-Mito were significantly lower than that in ER-Mito (Figure 5G). In contrast, the distribution of Aβ42 at these four CSs reached 68.8%, 46.0%, 64.1%, and 60.5%, respectively, and there was a particularly remarkable increase in GO-Lyso, where its proportion was significantly higher than that in GO-Mito and comparable to those in ER-Lyso and ER-Mito (Figure 5H). Therefore, the different localization of APP and its amyloidogenic product Aβ42 on these CSs reinforces the implication of the GELM in APP amyloidogenic processing, suggesting a potential correlation between progressive CS modulation and the distribution patterns.

Furthermore, considering that abnormalities of dendritic spines are a significant hallmark of amyloid toxicity 61, dendritic spike structures in C3da neurons were assessed in APP amyloidogenic processing. The results showed that, similar to the disturbance pattern observed in the GELM CSs, there were progressive defects in the structural plasticity of dendritic spikes during APP amyloidogenic processing (Figure S6). Moreover, the association of the GELM network with these structural defects was explored by manipulating GELM regulators. Knockdown of Miro could rescue the density and dynamics of dendritic spikes in Aβ42 neurons, but did not change the length of spikes (Figure S6). These findings suggest that disturbances in the GELM CSs during APP amyloidogenic processing, may further lead to defects in the structural plasticity of dendritic spikes.

In summary, the GELM network is progressively disrupted during the amyloidogenic processing of APP, characterized by the upregulation in the density and motility of single- to multi-CSs, which is associated with defects in structural plasticity of dendritic spikes.

## Discussion

Dendritic organelles play crucial roles in dendrite development and neurodegenerative diseases, but their communication remains unclear, especially *in vivo* under physiological and pathological conditions. In this study, we established an *in vivo* model of multi-organelle contact network, namely the GELM network, to elucidate how organelle crosstalk is disrupted in APP amyloidogenic processing. In this model, four types of CSs were demonstrated in a network with multi-organelle contacts, and interactions among them were confirmed through the diverse complementary modulations in their density and motility, at network level and across cellular pathways. Furthermore, all four types of CSs were revealed to be disturbed, through a progressive pattern in APP amyloidogenic processing. Moreover, Miro was identified to play a role in Aβ42-induced perturbations in both the GELM network and dendrite structural plasticity.

The model proposes an interactive GELM network with multiple CSs coordination. The organization of multiple organelles has been characterized in cultured cells and* in vitro* tissues, and a few multi-organelle contacts have been observed. For example, the interactome of six organelles in COS-7 cells 31, and the ER-peroxisome-mitochondria complex in mouse hepatocytes 62. Moreover, multi-way contacts are also hinted at in dendrites through the frequent spatial overlaps of distinct CSs between ER and other organelles/plasma membrane (PM) as shown by focused ion beam-scanning electron microscopy 10. Here, we describe the organization of multiple organelles and their interactions under physiological conditions using a dendrite model of organelle contact. A GELM network dominated by multi-organelle contacts was emphasized. Moreover, the interactive modulations among multiple CSs support the interplay between organelles in this GELM contact network. For instance, the regulation of mitochondrial CSs by Miro or Porin2 also acted on non-mitochondrial CSs. Additionally, Miro caused the consistent density change of two mitochondrial CSs (GO-Mito and ER-Mito) suggesting the potential molecule connecting multi-organelle contacts. These findings collectively indicate that dendritic organelles are organized in a coordinated and interactive network of multi-organelle contacts.

Modulations in CS dynamics expand our understanding of the homeostatic coordination between organelle contacts. The reciprocal regulation between CSs indicates a homeostatic mechanism for maintaining cellular functions 63. These regulations have been evaluated based on the static structural characteristics of CSs, including their number and size. For instance, the number of two ER-related CSs (ER-PM and ER-peroxisome) exhibit opposite regulation when inhibiting NPC1 64. Here, the dynamic feature of four types of CSs was examined, by *in vivo* imaging of the CSs. The coordination between motility and density of these CSs was found. e.g., a decrease in the density of the GO-Mito CSs, while an increase in the motility of ER-Lyso CSs was demonstrated when knocking down vesicular transporter X11L. Meanwhile, there also existed complementary regulation in CS density alone mediated by tethers, such as Miro and Porin2. These findings expand the dynamics of CSs as a new feature for evaluating the coordination of inter-organelle communication, suggesting a novel pathway for maintaining homeostasis in dendrites.

Both top-down and parallel patterns of CS regulation are exhibited between vesicular transporters and CS tethers, indicating a complex relationship between inter-organelle communication via CSs and transport vesicles. Based on the previous studies, a top-down regulation pattern between them has been suggested, where vesicular transporters can alter CSs via direct interaction or regulating the trafficking of their tethers. For instance, vesicular SNARE proteins disrupt the formation of the ER-PM CSs, by interacting with the tether ORP/Osh 50. Inhibition of COPⅠ reduces the ER-Mito CSs by inducing the mislocalization of several tethers, including BAP31 and VAPB 51. Here, our results also suggest a parallel regulation pattern between vesicular transporters and the CS tethers, based on their distinct regulation roles in CS properties. In this study, the vesicular transporter AP-1γ was found to solely regulate CS motility. That is, AP-1γ-RNAi induced motility upregulation in two non-mitochondrial CSs (ER-Lyso and GO-Lyso). In contrast, the mitochondrial CS tethers, such as Miro and Porin2, as well as the non-mitochondrial CS tethers, including Sac1 65, 66 and VPS13 67, were only involved in the density regulation of these CSs (Figure S7). Notably, Sac1 and AP-1γ regulated the same types of CSs, but differed in their properties, highlighting the parallel regulation pattern. Thus, a complex interaction pattern is proposed between the two communication pathways: vesicular transporters and the CS tethers can work in both top-down and parallel patterns to regulate CSs.

The progressive disturbances of the CSs at the network level suggest a cascaded dysregulation of organelle crosstalk in APP amyloidogenic processing. The canonical amyloid cascade hypothesis, positing amyloid plaques to be the cause of AD, faces challenges. Pathological studies in AD patients and animal models have demonstrated a weak association between amyloid deposition and cognitive decline 68. Instead, soluble amyloid processing products, including soluble Aβ oligomers 69, 70 and APP-βCTFs 71, 72, have been identified as the cause of synaptic damage and memory impairment. Their cellular toxicity has been reported to originate from disturbances in the CSs, including the ER-Lyso CSs by APP-βCTFs 55, and the ER-Mito CSs by both APP-βCTFs 25 and Aβ42 26, 27. The interconnectivity of CSs has inspired systematic exploration of CSs dysregulation in AD pathology 34. Here, we investigated the impact of three states of APP amyloidogenic processing on an integrated CS network (the GELM network). Our results suggest that perturbations in organelle crosstalk during APP amyloidogenic processing may originate from a specific type of CSs, such as the ER-Lyso CSs, which are initially disturbed, and then spread throughout the GELM network as the amyloidogenic products form or accumulate. These findings provide a more comprehensive understanding about the cellular toxicity of amyloidosis and AD pathology.

A CS-related structural mechanism is suggested underlying the dendritic pathologies in AD. The loss of dendritic spines in mammals is a hallmark of amyloid toxicity, and is closely linked to synaptic dysfunction 61. These structural defects have been reported to be associated with the dysregulation of organelles, such as GOs 5, Lyso 73, 74 and Mito 75. Here, the mutual regulation between the GELM CSs and dendritic spikes in C3da neurons, was demonstrated by the specific regulators. Furthermore, the progressive perturbations in both the GELM network and dendritic spike structure were observed during APP amyloidogenic processing, and could be jointly rescued by the manipulation of the GELM network regulator, Miro. These findings suggest a potential mechanism underlying the cytotoxicity of APP amyloidogenic processing: the structural defects of dendritic spikes result from the disruption of the GELM network via the Miro-related pathway. Nevertheless, given the complexity of the inter-organelle communication network 34, a broader range of interactions in this toxicity mechanism still needs to be studied in the future.

## Conclusion

In conclusion, the homeostatic GELM network serves as an excellent model of inter-organelle crosstalk. By elucidating disturbances in the GELM CSs in APP amyloidogenic processing, we propose a potential working model: amyloidogenic products disrupt the organization of the GELM CSs in density and dynamics, leading to defects in the structural plasticity of dendritic spikes, which represent the dendritic degeneration in AD pathology. Furthermore, these perturbations in CSs and dendritic spikes can be partially rescued by the knockdown of Miro. This GELM network model provides a versatile tool for future research on related neurological disorders and the therapeutic strategies.

## Methods and Materials

### Transgenic lines constructed in this study

To generate organelle-targeted split-GFP probes, those are 10 × UAS-Lamp1-V5-FKBP-GFP_1-10_, 10 × UAS-GFP_1-10_-V5-FKBP-Lamp1, 10 × UAS-Tom20-V5-FKBP-GFP_1-10_, 10 × UAS-GFP_11_-HA-FRB-dGM130-∆N100 and 10 × UAS-GFP_11_-HA-FRB-Cb5, we amplified the following genes by PCR: *Lamp1* with FKBP- or V5-ligated termini from Lamp1-RFP, V5-FKBP and Tom20 from Tom20-V5-FKBP-AP_pLX304, dGM130-∆N100 from 10 × UAS-EGFP-dGM130-∆N100 76, Cb5 and HA-FRB from EX-HA-FRB-Cb5_pLX304, GFP_1-10_ with FKBP- or V5-ligated termini from paavCAG-post-mGRASP-2A-dTomato, and GFP_11_ from paavCAG-pre-mGRASP-mCerulean. To generate FRET probes of GO-Lyso, including 10 × UAS-Lamp1-V5-FKBP-mCherry, 10 × UAS-mCherry-V5-FKBP-Lamp1 and 10 × UAS-EGFP-HA-FRB- dGM130-∆N100, mCherry with FKBP- or V5- ligated termini were amplified from pACUH-GFP_11_ × 7-mCherry-α-tubulin, EGFP from pEGFP-N1. The 10 × UAS vector was obtained from the digestion of pJFRC2-10 × UAS-IVS-mCD8-GFP with *XhoⅠ* and *XbaⅠ*. Then, these inserted genes were fused to 10 × UAS vector, respectively.

To perform the four-color imaging of the GELM network, 10 × UAS-ManII-mTagBFP2, 10 × UAS-Mito-mCardinal were constructed. Firstly, *ManII*, mTagBFP2 and mCardinal were respectively amplified from constructs of ManII-TagRFP, PNCS-mTagBFP2-mClover3 and PNCS-mCardinal by PCR. *Mito* cDNA was amplified from UAS-Mito-GFP *Drosophila*. Then, we fused *ManⅡ* together with mTagBFP2 and *Mito* together with mCardinal to the10 × UAS vector.

10 × UAS-VSVG-EGFP were generated to label the structure of dendritic spikes. *VSVG* cDNA was amplified from Ub-VSVG::SP::GFP *Drosophila*. Then, *VSVG* and EGFP were inserted into 10 × UAS vector.

To generate 10 × UAS-Aβ42-TagRFP and 10 × UAS-mOrange2-APP, cDNA of *Aβ42* and *APP* were amplified from UAS-Aβ42 and UAS-APP695 *Drosophila*, and mOrange2 from construct of PNCS-mOrange2, and then they were fused to 10 × UAS vector, respectively.

Germline transformations on third chromosome or second chromosome were achieved by the injection of PBac{y[+]-attP-3B}VK00033 or P{CaryP}attP40 embryos.

### Confocal microscopy

For all live imaging, third-instar larvae were anesthetized with ether, and then mounted in halocarbon oil 700. To image the C3da neurons, the larvae were adjusted to the dorsal view. Finally, high vacuum grease was added around the larvae, and a coverslip was gently pressed flat above them. The C3da neurons located at A4-A6 segments were imaged.

Live images of CSs and dendritic spikes were acquired using an Olympus FV1000 confocal laser scanning microscope with a 60 × oil objective lens (NA = 1.42) and equipped with 405 nm, 488 nm, 543 nm and 633 nm lasers. To analyze the distribution and dynamics of CSs labelled with split-GFP or FRET probes, the reconstituted GFP and FRET signals were excited with 488 nm laser and collected 500-530 nm and 599-699 nm, respectively. Images were captured in Z-stack mode with 0.02 × 0.02 × 0.5 μm^3^ voxel for soma and 0.10 × 0.10 ×1 μm^3^ voxel for dendrites. Time-lapse images were acquired at 6 s/frame for 101 frames with a XY resolution of 0.21 × 0.21 μm. In the imaging of GOs, lysosomes and their CSs, ManⅡ-mTagBFP2, LAMP1-mCherry and reconstituted GFP were in two groups (G1: mTagBFP2 and mCherry, G2: reconstituted GFP) that were sequentially excited and collected. Time-lapse images were acquired at 10.6 s/frame with a XY resolution of 0.14 × 0.14 μm. To estimate the dynamics of dendritic spikes, time-lapse images of dendritic spikes were recorded with an XY resolution of 0.21 × 0.21 μm, and at an interval of 17 s.

To visualize the organization of four organelles with a super spatial resolution, we performed the four-color Airyscan imaging on a Zeiss LSM900 Airyscan 2 confocal microscope with a 63 × oil objective lens (NA = 1.40). Four fluorophores (mTagBFP2, GFP, RFP and mCardinal) were excited with 405 nm, 488 nm, 561 nm and 639 nm lasers, and collected at 425-470 nm, 500-530 nm, 571-600 nm and 630-700 nm. Images were collected with Airyscan GaAsP-PMT detector and images of the soma and dendrites were acquired with a XY resolution of 0.04 × 0.04 μm.

To visualize the dynamic of four organelles in dendrites, four-color live imaging of organelles was acquired on Nikon AX laser confocal scanning microscope. Images were obtained with a XY resolution of 0.20 × 0.20 μm and Z-stacks of three slices. Time-lapse images were acquired at 6 s/frame for 10 min with a Nikon Perfect Focus System (PFS).

### Image processing

#### Analysis of dynamic CSs

To describe the characteristics of CSs, time-lapse images of organelles were analyzed. Images were first deconvoluted using Huygens 23.04 software. The CSs within dendrites about 100 μm from the soma and second order of dendritic branches were analyzed. The density was analyzed from the first frames, and the motility was analyzed from the continuous time-lapse images by using kymograph. To obtain the kymograph, three steps were performed on these image stacks by Fiji, including straightening the dendrites, reslicing and z-stacking to obtain kymographs. The mobile CSs were defined as one which moved more than 0.5 μm in any direction.

#### The structural plasticity of dendritic spikes

The structural plasticity of dendritic spikes was quantified in terms of the density, length and dynamics of spikes. Density and length were analyzed from the first frames of time-lapse images of dendritic spikes. To analyze the dynamics of spikes, we generated temporal projection images from time-lapse images using the “Temporal-Color Code” tool in Fiji by applying a red-green-blue lookup-table (LUT). The dynamics of spikes were then determined by the percentages of extensions, retractions, and both.

#### Analysis of the organelle organization patterns

To analyze the organization of the four organelles, we first identified their spatial overlap through line scan analysis. The four-color images were first performed the Airyscan processing using Zen 3.1 software. Then, fluorescence intensity line scans were performed using Fiji software (NIH, USA) by drawing a line across the center of the dendrite, which allowed us to assess contacts as the overlap in fluorescence intensities among the GOs, ER, Lyso and Mito.

In the analysis of spatial organization patterns of the four organelles, we calculated the number of organelles without overlapping and in complexes of two-, three-, and four-organelles in the snap images. To assess the stability of the organization, the four-color time-lapse imaging was processed and used to generate kymographs with merged channels. Then, we counted the number of overlaps between all the six organelle pairs (GO-Lyso, GO-ER, GO-Mito, ER-Lyso, ER-Mito, Lyso-Mito) at 2-min intervals (i.e., at time points of 0, 2, 4, 6, 8, and 10 min) during the 10 min time-lapse imaging.

### Statistical analysis

Comparative analysis among multiple groups was performed using one-way ANOVA, followed by Tukey or Dunnett post-hoc tests in Prism 8 (GraphPad) software. Comparisons between two groups were performed using unpaired Student's t-test. Bar graphs are presented as mean ± SEM.

## Supplementary Material

Supplementary figures.

## Figures and Tables

**Table 1:** Key resources table

REAGENT OR RESOURCE	SOURCE	IDENTIFIER
**Experimental models: Organisms/strains**		
GAL4^19-12^	Ref. 77	N/A
UAS-ManII-GFP	Ref. 78	N/A
UAS-ManII-TagRFP	Ref. 4	N/A
UAS-HRP-DsRed	Ref. 79	N/A
UAS-KDEL-RFP	BDSC	BDSC 30909
UAS-Lamp1-GFP	BDSC	BDSC 42714
UAS-Mito-GFP	BDSC	BDSC 8442
UAS-Rac1	BDSC	BDSC 28874
UAS-Miro	BDSC	BDSC 51646
Miro-RNAi	Tsinghua Fly Center	THU4782
Porin2-RNAi	Tsinghua Fly Center	THU2090
Sac1-RNAi	Tsinghua Fly Center	TH03579
VPS13-RNAi	Tsinghua Fly Center	TH03579
Porin-RNAi	Tsinghua Fly Center	TH03163
AP-1γ-RNAi	Tsinghua Fly Center	THU2696
X11L-RNAi	Tsinghua Fly Center	THU2492
Rab5-RNAi	Vienna Drosophila Resource Center	V34096
Rab6-RNAi	Tsinghua Fly Center	THU2652
δ-COP-RNAi	Tsinghua Fly Center	THU3459
ζ-COP-RNAi	Tsinghua Fly Center	THU3495
UAS-APP695	BDSC	BDSC 6700
UAS-Aβ42	BDSC	BDSC 33769
UAS-BACE, UAS-APP.695	BDSC	BDSC 33797
Ub-VSVG::SP::GFP	Pastor lab	N/A
**Bacterial and virus strains**		
Lamp1-RFP	Addgene	Addgene plasmid # 1817
Tom20-V5-FKBP-AP_pLX304	Addgene	Addgene plasmid # 120914
EX-HA-FRB-Cb5_pLX304	Addgene	Addgene plasmid # 120915
paavCAG-post-mGRASP-2A-dTomato	Addgene	Addgene plasmid # 34912
paavCAG-pre-mGRASP-mCerulean	Addgene	Addgene plasmid # 34910
pACUH-GFP_11_ × 7-mCherry-α-tubulin	Addgene	Addgene plasmid # 70218
pJFRC2-10 × UAS-IVS-mCD8-GFP	Addgene	Addgene plasmid # 26214
dGM130-∆N100	Ref. 76	N/A
PNCS-mTagBFP2-mClover3	Chu lab	N/A
PNCS-mCardinal	Chu lab	N/A
PNCS-mOrange2	Chu lab	N/A

## Abbreviations

AD: Alzheimer's disease
CS: contact site
APP: amyloid precursor protein
APP-CTFs: C-terminal fragments of amyloid precursor protein
Aβ: β-amyloid peptide
GELM: the contact network among Golgi outposts, the endoplasmic reticulum, lysosomes, and mitochondria
GOs: Golgi outposts
Mito: mitochondria
ER: the endoplasmic reticulum
Lyso: lysosome
PD: Parkinson's disease
C3da neurons: the class Ⅲ dendritic arborization neurons
FRET: fluorescence resonance energy transfer
RNAi: RNA interference
NA: numerical aperture
LUT: lookup-table
