# Supplementary material for: The inter-organelle cross-talk finely orchestrated in the amyloidogenic processing of amyloid precursor protein in dendritic arborization neurons of Drosophila
Source: Theranostics. 2025 Feb 10;15(7):2951–66. doi: 10.7150/thno.104345 (PMC11898278; doi:10.7150/thno.104345)
Supplement: Supplementary file 1 — Supplementary figures. [file thnov15p2951s1.pdf]

Supplementary information, Figure S1

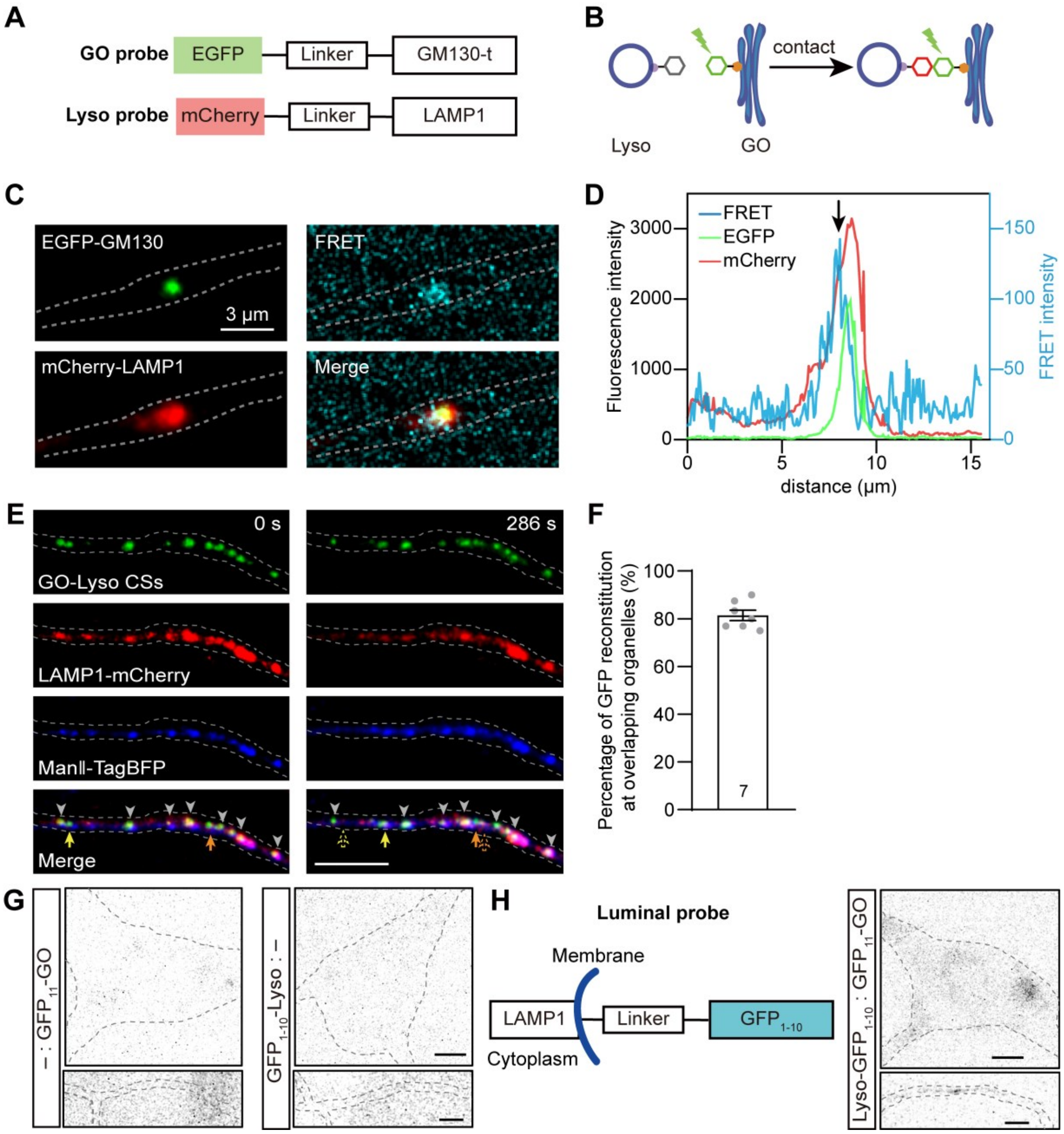

Figure S1 Check the accuracy of the split-GFP probes using for labeling CSs in dendrites. (A-D) The detection of GO-Lyso CSs with the alternative FRET probes. (A) Schematic diagram of the GO and Lyso FRET probes. (B) Cartoon showing the detection of GO-Lyso CSs using the FRET probes. (C) Confocal images showing the FRET signal (cyan) colocalized with GO (green) and Lyso (red) in dendrite expressing the complementary GO and Lyso FRET probes. (D) Fluorescence intensities of the three channels in the dendritic region in (c). Arrow indicates the site of FRET signal. (E, F)

Check the position of the reconstructed GFP. (E) Three-color time-lapse images showing the co-localization and co-movement of split-GFP probes labelled CSs with two organelles (GOs: ManII-mTagBFP2, Lyso: Lamp1-mCherry). Arrowheads indicate the co-localized contact sites with organelles; the yellow arrows indicate co-movement. (F) Quantitative analysis of the proportion of GFP reconstitution at two overlapped organelles labelled with fluorescent proteins. The number in the bar diagram represents the sample size from three *Drosophila* larvae. Data are the means ± SEM. (G) Representative images showing that no apparent GFP signal was detected when GFP_{11} or GFP_{1-10} were expressed alone. (H) Leaking test of split-GFP probes through co-expressing the luminal lysosomal probe (left) and GO probe in C3da neurons showing no reconstituted GFP puncta were detected (right). Scale bar: 3 μm in (C), 2 μm in soma and 10 μm in dendrites in (E), (G) and (H).

Supplementary information, Figure S2

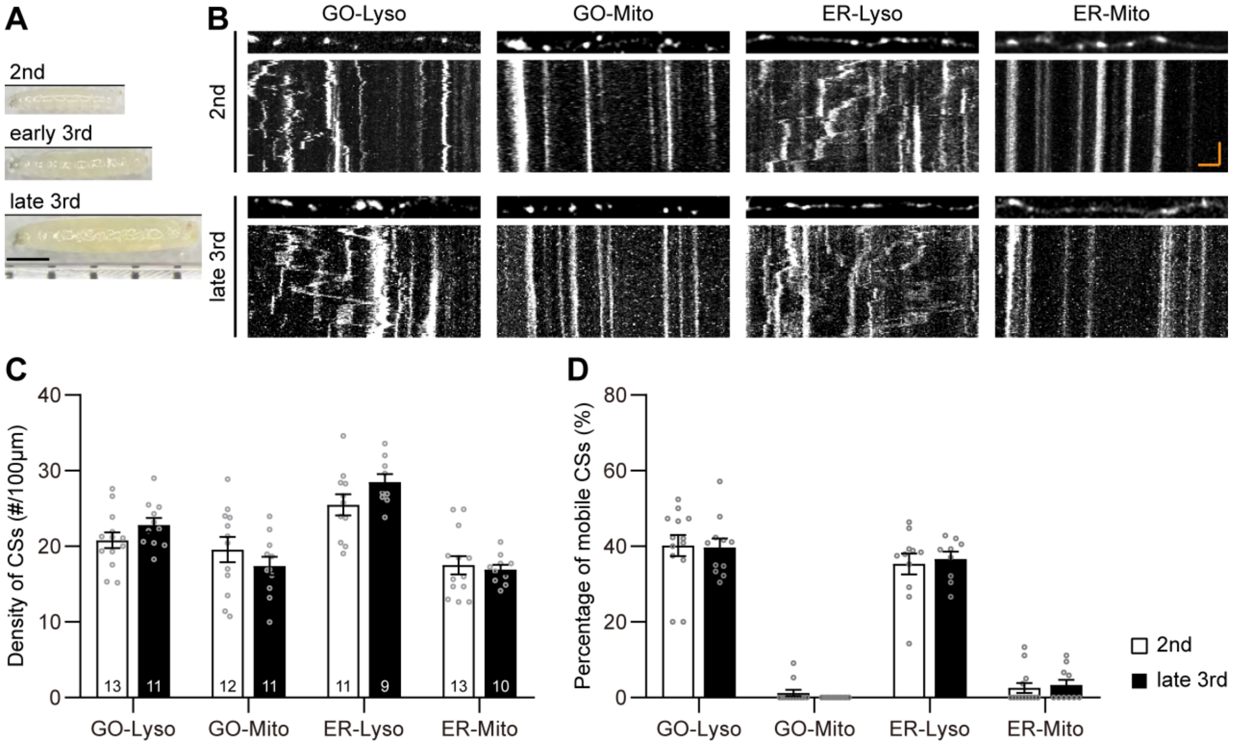

Fig. S2 Organization patterns of the four CSs in dendrites during larva development. (A) Photographs of larvae at different ages. (B) Representative confocal images and the corresponding kymographs showing the distribution and movement of four types of the GELM CSs in second (upper) and late third (bottom) instar larvae. (C and D) Quantitative analysis of the CS density (C) and motility (D) in larvae at different ages. The numbers in the bar diagram represent the sample sizes of each experimental group from three to six *Drosophila* larvae. For all quantifications, data are the means ± SEM. Unpaired Student's t-test in (C and D) showing no significant difference. Scale bars: 1 mm in (A); horizontal scale bar: 4 μm and vertical bar: 2 min in (B)

Supplementary information, Figure S3

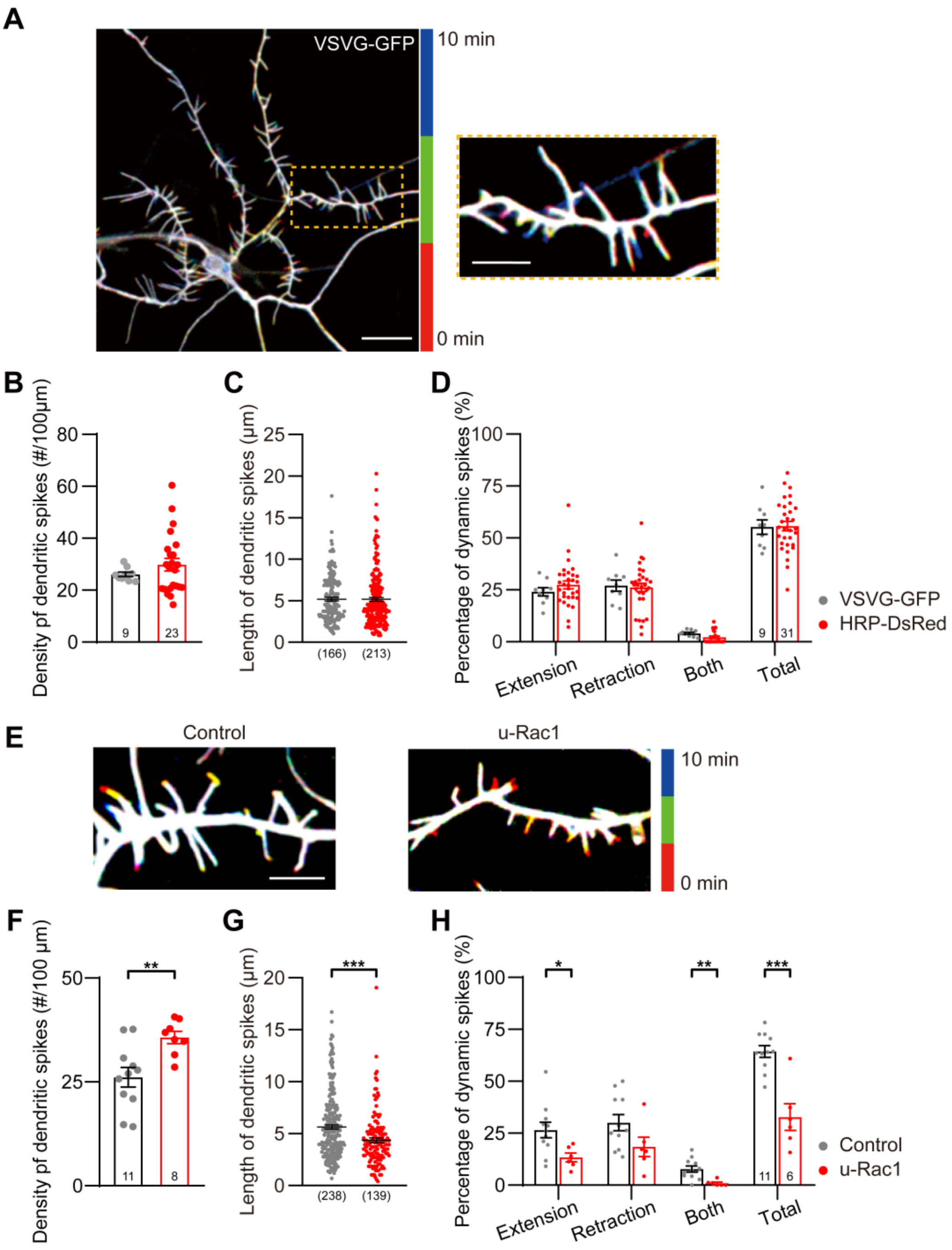

Figure S3 The ectopic expression of Rac1 alters the structural plasticity of dendritic spikes. (A) Temporal-code images from time-lapse imaging of dendritic spikes labeled with VSVG-GFP for 10 min. The area of the dotted box in (left) is enlarged in (right). Scale bars in left: 20 μm, right: 10 μm. (B-D) Comparison of the structural plasticity of dendritic spikes labeled with VSVG-GFP to that labeled with HRP-DsRed. Density in (B), length in (C) and dynamics in (D). (E) Temporal-code

images from time-lapse imaging showing the dynamic dendritic spikes under the normal condition and the ectopic expression of Rac1. (F-H) Quantitative analysis of the alteration in structural plasticity of dendritic spikes by Rac1. Density in (F), length in (G) and dynamics in (H). The temporal color codes are red-green-blue in (A) and (E). The numbers in the bar diagrams represent the sample sizes of each experimental group from three to seven *Drosophila* larvae. For all quantifications, data are the means ± SEM. Unpaired two-sided Student's t-test in (B-D) and (F-H). *p < 0.05, **p< 0.01, ***p < 0.001. Scale bars: 20 μm in (A, left), 10 μm in (A, right) and (E).

Supplementary information, Figure S4

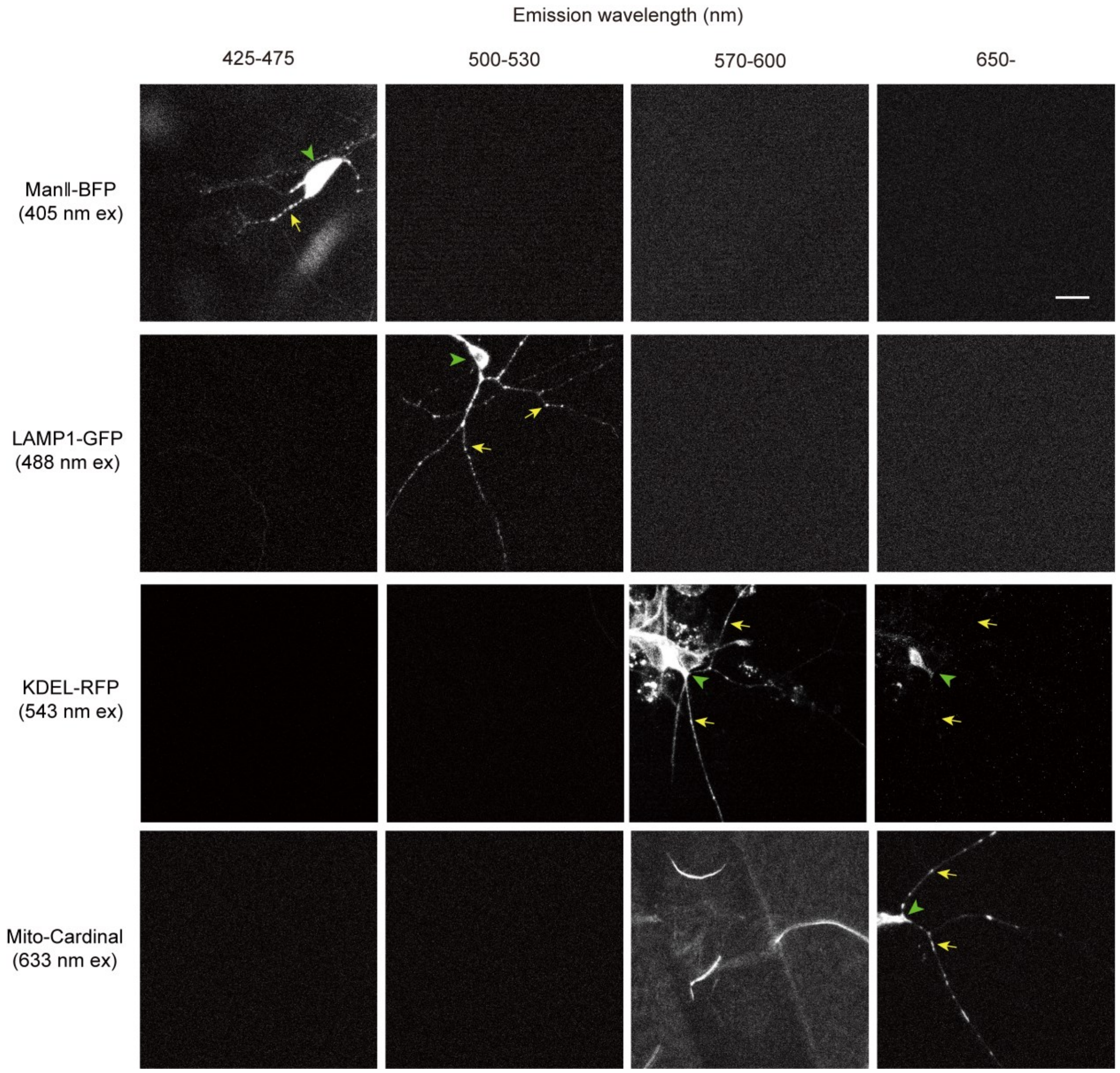

Figure S4 The range of emission wavelengths of the four fluorescent proteins labelling organelles. Confocal images of singly labelled neurons were recorded at the four wavelength ranges upon the excitation with 405 nm, 488 nm, 543 nm and 633 nm, respectively. Green arrowheads indicate soma and yellow arrows indicate dendrites. Scale bar: 15 μm.

Supplementary information, Figure S5

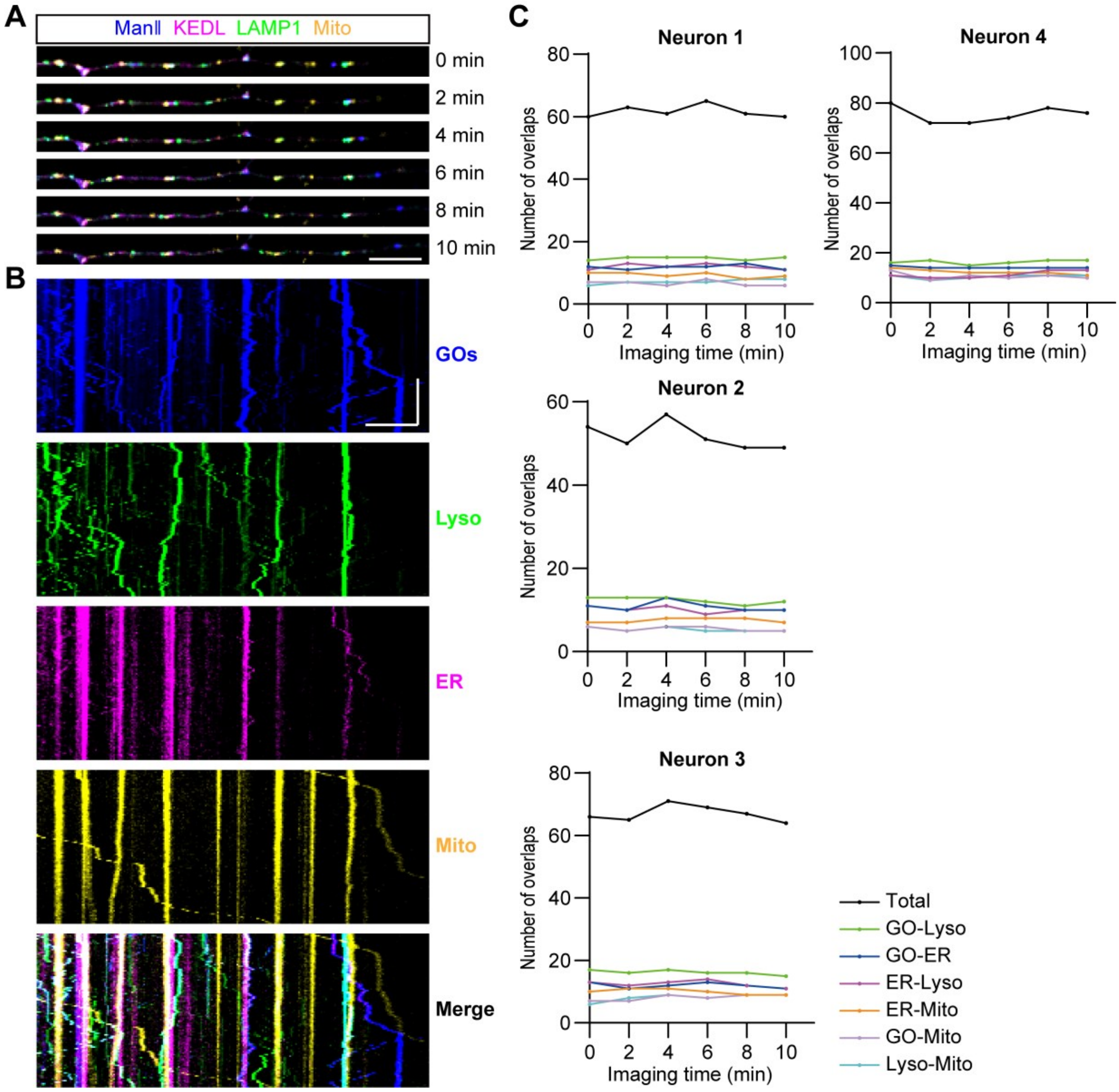

Figure S5 Dynamic but stable numbers of contacts between dendritic GOs, ER, Lyso and Mito over time. (A, B) Representative confocal images of dendrite expressing the four organelle markers at six time points (0, 2, 4, 6, 8 and 10 min) (A) and the corresponding kymographs (B) obtained from time-lapse imaging show the dynamic contacts between the four organelles. GOs, ER, Lyso and Mito were labelled with ManII-mTagBFP2, KDEL-RFP, LAMP1-GFP and Mito-mCardinal, respectively. (C) Numbers of the total contacts and contacts between each organelle pair in dendrites of four neurons

over 10 min. Analysis of their coefficients of variation showed that they were all not exceeding 15%, and the average was 7.9%. Horizontal scale bar: 10 μm and vertical bar: 3 min.

Supplementary information, Figure S6

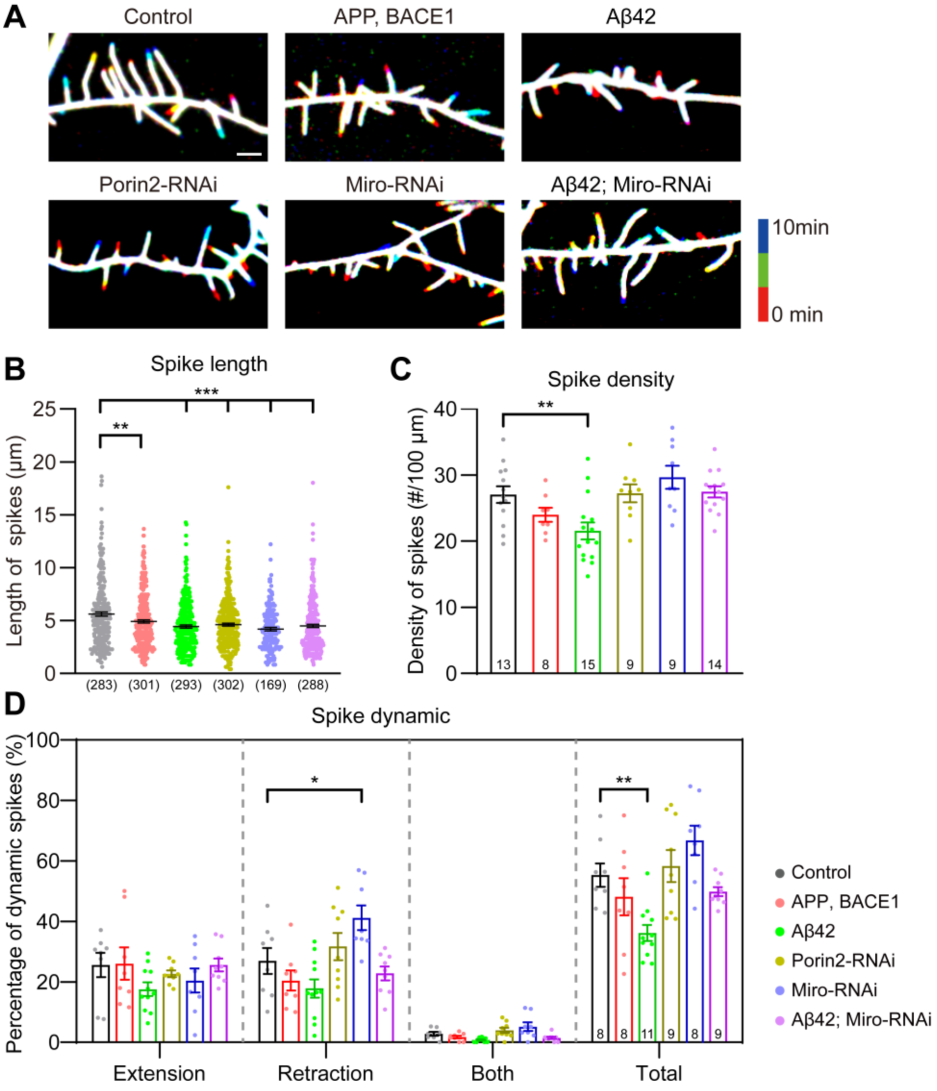

Figure S6. The defects and rescue in structural plasticity of dendritic spikes in APP amyloidogenic processing. (A) Representative images showing the dynamic dendritic spikes under different conditions: normal, β-APP, and Aβ42 conditions (upper panel); and the manipulation of the CS tethers in normal and Aβ42 neurons (bottom panel). The dendritic spikes were labelled with VSVG-GFP, and their dynamics in 10 min were demonstrated by using temporal-code LUT of red-green-blue. (B-D) Quantitative analysis of the structural plasticity of dendritic spikes. Length in (B), density in (C), and dynamic in (D). The numbers in the bar diagrams represent the sample sizes of each experimental group from three to six *Drosophila* larvae. For all quantifications, data are the

means ± SEM. One-way ANOVA multiple comparisons test with Dunnett correction in (B-D). $*p < 0.05$, $**p < 0.01$, $***p < 0.001$. Scar bar: 5 μm in (A)

Supplementary information, Figure S7

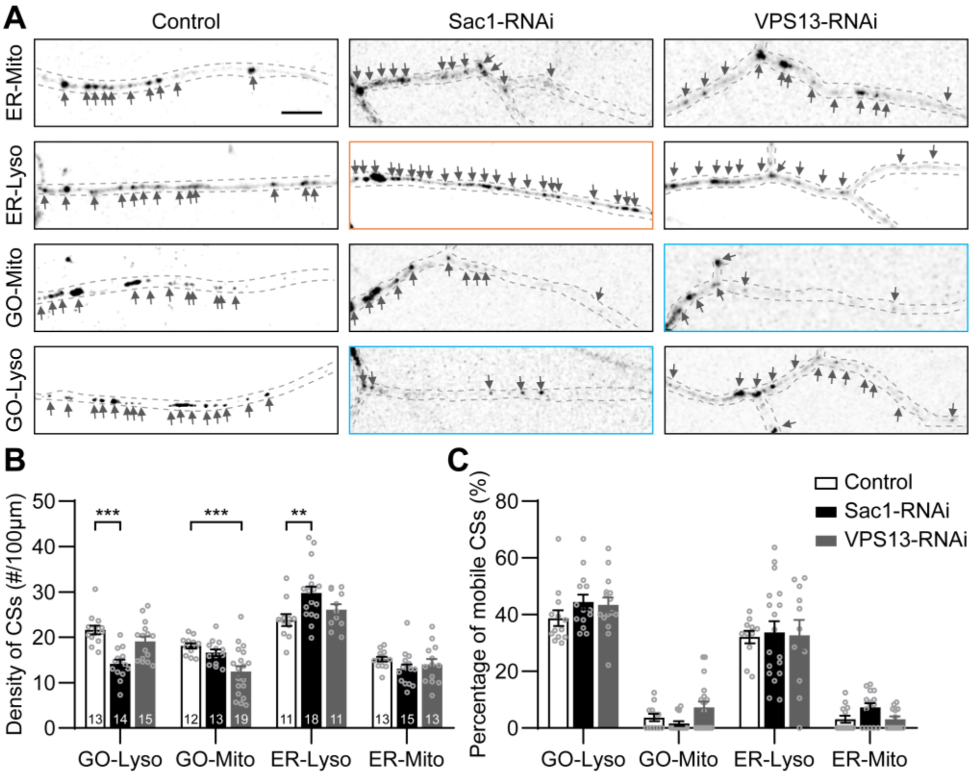

Fig. S7 Modulations of the GELM CSs by non-mitochondrial CS tethers. (A) Representative confocal images of four types of the GELM CSs in wild-type neurons and neurons with knockdown of the CS tethers Sac1 and VPS13. The CSs were labelled by split-GFP probes, and indicated with arrows. Dendrites in blue boxes show decreases in CS density and orange ones show the increases. (B, C) Quantitative analysis of the densities (B) and motilities (C) of four types of CSs. The numbers in the bar diagram represent the sample sizes of each experimental group from four to seven *Drosophila* larvae. For all quantifications, data are the means ± SEM. One-way ANOVA multiple comparisons test with Dunnett correction in (B, C). $^{**}p < 0.01$, $^{***}p < 0.001$. Scar bar: 10 μm in (A)
